# The complete mitochondrial genome of the common dace *Leuciscus leuciscus* (Teleostei: Leuciscidae)

**DOI:** 10.1080/23802359.2025.2460779

**Published:** 2025-02-02

**Authors:** Vincent Haÿ, Richard Pividori, Agnès Dettai, Bo Delling, Gaël P. J. Denys

**Affiliations:** a Institut de Systématique, Evolution, Biodiversité, ISYEB – UMR 7205 – Sorbonne Université, CNRS, MNHN, UPMC, EPHE, Paris, France; b UAR Patrimoine Naturel – Centre d’expertise et de données (2006 OFB – CNRS – MNHN – IRD), Muséum national d’Histoire naturelle, Paris, France; c Department of Zoology, Swedish Museum of Natural History, Stockholm, Sweden; d UMR Biologie des organismes et écosystèmes aquatiques (BOREA 8067), Sorbonne Université, MNHN, CNRS, IRD, UCN, UA, Paris, France

**Keywords:** Leuciscidae, *Leuciscus leuciscus*, mitochondrion, phylogenetic

## Abstract

This study presents, for the first time, the complete mitochondrial genome of the Common Dace (*Leuciscus leuciscus* (Linnaeus 1758)), in association with a voucher specimen from the Swedish Natural History Museum. The complete mitogenome length is 16,603 bp and comprises 13 protein-coding-genes (PCGs), 22 tRNAs, 2 rRNAs, and one non-coding control region. A maximum likelihood phylogenetic reconstruction based on 13 PCGs of Leuciscids species confirm the monophyly of the *Leuciscus* genus. Furthermore, this reconstruction has corroborated the placement of other *Leuciscus* species in this genus as *L. leuciscus* is the type species of this genus.

## Introduction

*Leuciscus* Cuvier, 1816 is a freshwater genus of Leuciscidae represented by 18 species in Eurasia with valid names (Fricke et al. 2024). In recent years, the nomenclature and taxonomy of this genus have undergone significant changes, particularly as a result of the use of genetic data (e.g. Berg 1949; Lelek 1987; Kottelat 1997, 2006; Kottelat & Freyhof 2007; Perea et al. 2010). Among these changes is the common dace *Leuciscus leuciscus* (Linnaeus 1758), a septentrional cryophilic species with a widespread distribution from Western Europe to the Ural (Kottelat & Freyhof 2007), but invasive in Ireland (Caffrey et al. 2007). In Southwest France, three *Leuciscus* species names have been revalidated according to morphological criteria, molecular data, biogeographical events, and the evolutionary species concept *sensu* Wiley & Mayden (2000) (Costedoat et al. 2006; Kottelat & Freyhof 2007; Denys et al., 2024): *Leuciscus bearnensis* (Blanchard 1866), *Leuciscus burdigalensis* Valenciennes *in* (Cuvier and Valenciennes 1844) and *Leuciscus oxyrrhis* (La Blanchère, 1873). Some authors have also discussed the existence of a *Leuciscus* complex (Costedoat et al. 2006; Dubut et al. 2009). Hinsinger et al. (2015) already sequenced and published the complete mitochondrial genome of *L. burdigalensis* and *L. oxyrrhis,* however, the mitogenome of the most widespread species is not available.

Here we provide the mitogenome of a voucher specimen of *Leuciscus leuciscus* from the Swedish Natural History Museum, obtained by a double-multiplexing approach (Hinsinger et al. 2015; Denys et al. 2020). As *L. leuscicsus* has been defined as the type species of the genus *Leuciscus* (Cuvier 1816), the acquisition of a reference mitogenome for *L. leuciscus* is therefore useful for resolving phylogenetic studies within the *Leuciscus* complex or the Leuciscids (Miya and Nishida 2015), and as a reference for monitoring this species using environmental DNA (Schroeter et al. 2020).

## Material and methods

**Figure 1.**
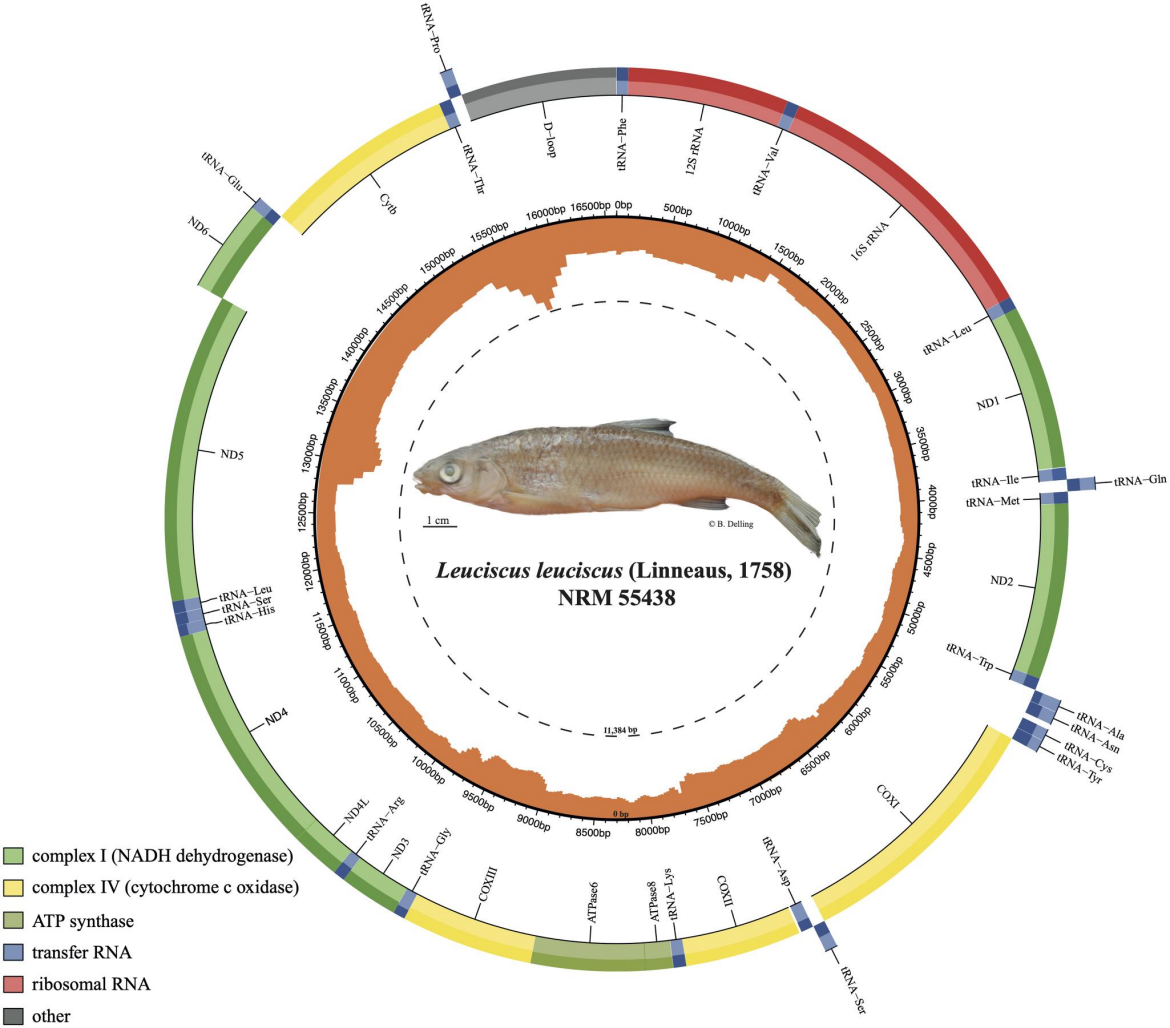
Circular map of the assembled *Leuciscus leuciscus* mitogenome (GenBank Accession: PQ059854) consisting of 13 protein-coding-genes (light green, dark green, and yellow), 22 tRNAs genes (blue), two rRNA genes (red) and one non-coding control region (grey). Genes encoded on the reverse and forward strands are depicted on the outside and inside of the circle, respectively. Read coverage is represented on the inner orange circle. Accession number PQ059854. Photo: B. Delling ©.

The voucher specimen is stored at the Swedish Museum of Natural History, cataloged NRM55438 (Figure 1) (https://artedi.nrm.se/nrmfish/, curator: Bo Delling, bo.delling@nrm.se). This specimen was caught by C. Aberg with electrofishing in the Hållsdammsbäcken Creek in the Göta Älv River (57°53’27”N, 12°02’35”E) in Sweden on 30 August 2006. It was received frozen to NRM and sampled for tissue before fixation in formalin and subsequent transfer to 70% ethanol. The tissue sample is a piece of muscle stored in 95% ethanol, −80 °C. This *specimen* has previously been barcoded for the mitochondrial *COI* gene (Ericson et al. 2020).

Total DNA was extracted from a piece of muscle using the Macherey and Negel NucleoSpin^®^ Tissue Kit following the manufacturer’s instructions on an Eppendorf EpMotion5075.

The complete mitochondrial genome (mtDNA) was amplified and sequenced using a protocol established by Hinsinger et al. (2015) with long PCR amplifications and a double-multiplexing approach. The mtDNA was amplified in three overlapping fragments (MT1, MT2 and MT3), using three pairs of primers (Table 1). A Hot Start LongAmpTaq DNA Polymerase (NewEngland Biolabs) modified protocol was used. Amplifications were performed by PCR in a final 18 µL volume including 5X LongAmpTaq Reaction Buffer, 0.4 ng/µL bovine serum albumin, 3.5% DMSO, 300 nmol/L of each primer, 300 µL/L of dNTPs, 0.9 unit of LongAmpTaq polymerase. After initial denaturation of 30s at 94 °C, the DNA was amplified through 45 cycles (20s-94 °C, 30s-62.5 °C and 15 min-65 °C) with a terminal elongation for 15 min at 65 °C on a Bio-Rad C1000 Touch Thermal Cycler. Successful PCRs were selected on ethidium-bromide stained agarose gels.

**Table 1.** Primers used for the amplification of the *Leuciscus leuciscus* mitogenome.

Fragment amplified	Length (bp)	Primers	Sequence (5’-3’)	Reference
MT1	6 500	12SL1091	AAACTGGGATTAGATACCCCACTAT	Kocher et al. 1989
		LeuMtH7061	GGGTTATGTGGCTGGCTTGAAAC	This study
MT2	6 800	L5231Leu	TAGATGGGAAGGCCTCGATCCTA	This study
		H11944Leu	CATAGCTTYYGCTTGGAYTTGCACCA	
MT3	5 200	12SH1478	TGACTGCAGAGGGTGACGGGCGGTGTGT	Hinsinger et al. 2015
		LeuMtL11910	CAGCTCATCCATTGGTCTTAGGAAC	This study

Sequencing was performed on Illumina^®^ MiSeq at the Concarneau marine station of the Muséum national d’Histoire naturelle (MNHN), using 250 paired-end standard V2. The mtDNA was assembled with Geneious Prime^®^ 2024.0.5 (www.geneious.com) by mapping trimmed paired reads to a reference sequence (*Leuciscus idus* (Linnaeus 1758), GenBank acc CM038646), and carefully quality controlled by eye along their whole length. The whole mtDNA was annotated and visualized through MitoAnnotator (Zhu et al. 2023) and Chloroplot software (Zheng et al. 2020).

236,272 reads (average length: 219.6) were assembled from the sequencing of the three long PCRs. Reads coverage throughout the entire sequence ranges from 371 to 11,384, with a mean coverage of 3202.2 (Supplementary Figure S1).

### Phylogenetic reconstruction

Mitogenomes from six species of *Leuciscus, Leuciscus baicalensis* (Dybowski 1874) (NC_024528), *Leuciscus waleckii* (Dybowski 1869) (MN105127 and NC_018825), *Leuciscus oxyrrhis* (NC_029425), *Leuciscus burdigalensis* (NC_029426), *Leuciscus idus* (CM038646 and MT584106), *Leuciscus merzbackeri* (Zugmayer 1912) (ON457584 and OR992052), as well as other members of the Leucicsinae sensu Tan and Armbruster (2018), Rutilus rutilus (Linnaeus 1758) (NC_068671 and OM736800), Abramis brama (Linnaeus 1758) (MT410936 and NC_020356), Blicca bjoerkna (Linnaeus 1758) (NC_020355) and Vimba melanops (Heckel 1837) (NC_031539), were retrieved from GenBank.

MtDNA sequences were aligned with MAFFT alignment (Katoh et al. 2002). The 13 protein-coding-genes (PCGs) were extracted and were used to reconstruct the maximum-likelihood phylogeny based on 13 PCGs from 11 species of Leuciscids. Tree was rooted with representatives of Phoxininae *sensu* Tan & Armbuster and Gobionids. For this purpose, species such as *Phoxinus phoxinus* (Linnaeus 1758) (NC_06867), *Pseudorasbora parva* (Temmick and Schlegel 1846) (KJ135626 and NC_015614) and *Gobio gobio* (Linneaus 1758) (AB239596 and OX442399) were used as outgroups. JModelTest (Darriba et al. 2012) was used to estimate the best evolutionary model and was GTR+I + G for both Akaike and Bayesian information criterion. Phylogenetic tree was inferred by Maximum Likelihood using RAxML-HPC2 on ACCESS (version 8.2.12) (Stamatakis 2014) with the previously inferred model and 1000 bootstrap iterations on the CIPRES Science Gateway (Miller et al. 2010) online platform.

## Results

*Leuciscus leuciscus* has a 16,603 bp-long mitogenome, with 13 PCGs (*ND1–6, NDL4, COXI–III, ATPase6, ATPase8* and *Cytb*), 22 transfer RNA (tRNA) genes, two ribosomal RNA (rRNA) (12S and 16S), and one control region (D-loop) (Figure 1) which follow the standard vertebrate order. Of the 13 PCGs, with the exception of *COXI*, which starts with GTG, the ATG codon is the predominant start codon. Seven PCGs (*ND2, COXII, ATPase6, COXIII, ND3, ND4,* and *Cytb*) have incomplete stop codons while the six other genes (*ND1, COXI, ATPase 8, NDL4, ND5* and *ND6*) terminate with canonical stop codon TAA or TAG (Table 2). There are seven overlapping genes (from 1 to 7 bp) and 12 intergenic spacers (from 1 to 32 bp). The nucleotide composition of the entire mitogenome was 28.7% (4772) for adenine, 26.4% (4379) for thymine, 27.0% (4481) for cytosine, and 17.9% (2971) for guanine. There are no repeated sequences in the D-loop.

**Table 2.** Gene composition of the complete mitochondrial genome of *Leuciscus leuciscus* detailing position, length, codon start and stop, and direction.

Fragment	First nucleotide	Last nucleotide	Length (bp)	Start codon	Stop Codon	Direction
tRNA-Phe	1	69	69			Forward
12S rRNA	70	1027	958			Forward
tRNA-Val	1028	1099	72			Forward
16S rRNA	1100	2790	1691			Forward
tRNA-Leu	2791	2866	76			Forward
ND1	2867	3841	975	ATG	TAA	Forward
tRNA-Ile	3846	3917	72			Forward
tRNA-Gln	3916	3986	71			Reverse
tRNA-Met	3988	4056	69			Forward
ND2	4057	5101	1045	ATG	T-	Forward
tRNA-Trp	5102	5172	71			Forward
tRNA-Ala	5174	5242	69			Reverse
tRNA-Asn	5244	5316	73			Reverse
tRNA-Cys	5349	5415	67			Reverse
tRNA-Tyr	5417	5487	71			Reverse
COXI	5489	7039	1551	GTG	TAA	Forward
tRNA-Ser	7040	7110	71			Reverse
tRNA-Asp	7114	7187	74			Forward
COXII	7201	7891	691	ATG	T-	Forward
tRNA-Lys	7892	7967	76			Forward
ATPase8	7969	8133	165	ATG	TAG	Forward
ATPase6	8127	8809	683	ATG	TA-	Forward
COXIII	8810	9593	784	ATG	T-	Forward
tRNA-Gly	9594	9665	72			Forward
ND3	9666	10,014	349	ATG	T-	Forward
tRNA-Arg	10,015	10,084	70			Forward
NDL4	10,085	10,381	297	ATG	TAA	Forward
ND4	10,375	11,756	1382	ATG	TA-	Forward
tRNA-His	11,757	11,825	69			Forward
tRNA-Ser	11,826	11,894	69			Forward
tRNA-Leu	11,896	11,968	73			Forward
ND5	11,969	13,804	1836	ATG	TAG	Forward
ND6	13,801	14,322	522	ATG	TAG	Reverse
tRNA-Glu	14,323	14,391	69			Reverse
Cytb	14,396	15,536	1141	ATG	T-	Forward
tRNA-Thr	15,537	15,608	72			Forward
tRNA-Pro	15,608	15,677	70			Reverse
D-loop	15,678	16,602	925			Forward

**Figure 2.**
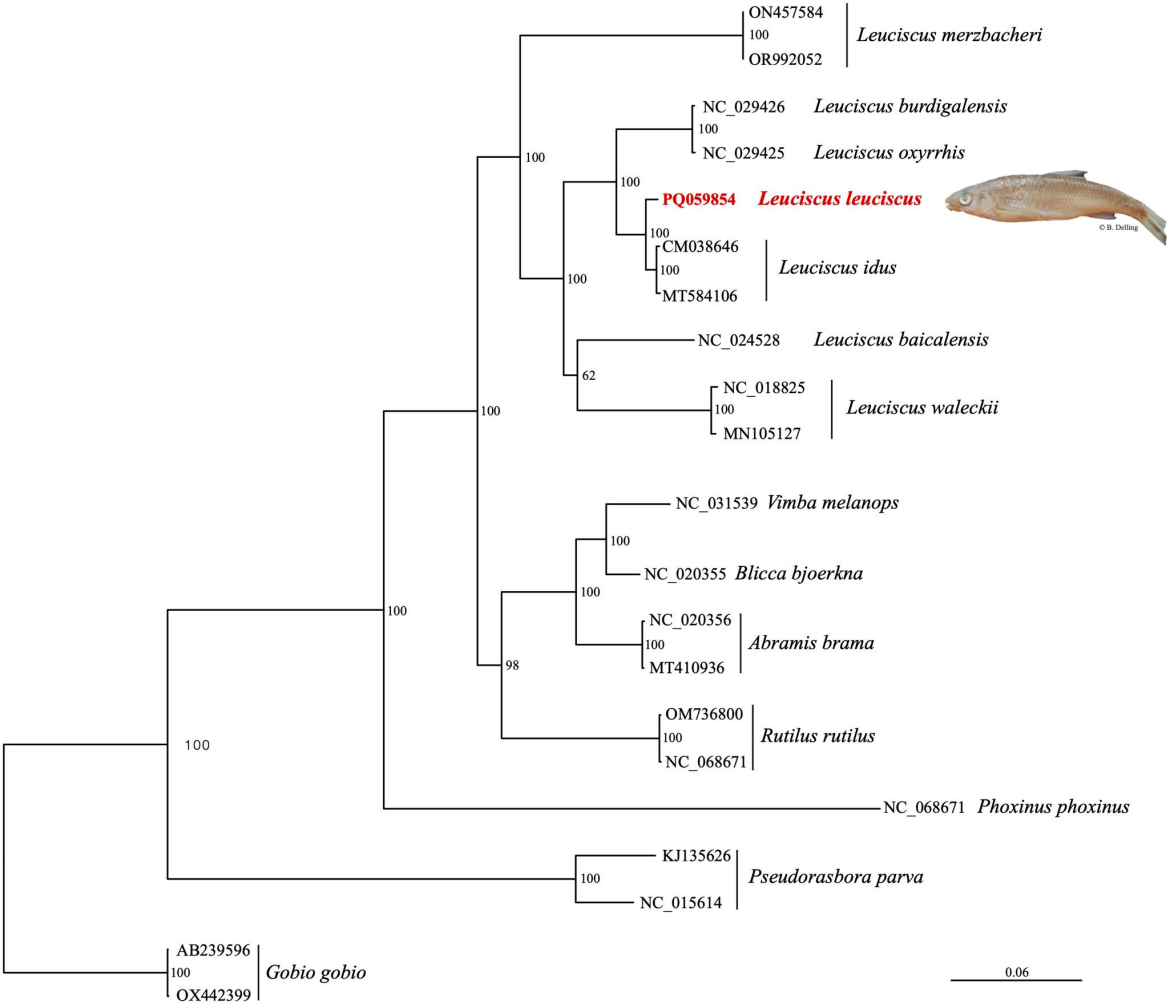
Maximum-likelihood (ML) phylogenetic tree based on the mitogenome of Leuciscus leuciscus (in red, accession number PQ059854) and other Leuciscinae species. Numbers at the nodes indicated bootstrap support values from 1000 replicates. The following sequences were used: *L. merzbacheri* acc ON457585 (unpublished), acc OR992052 (unpublished); *L. baicalensis* acc NC_024528 (Hu et al. 2015); *L. waleckii* acc NC_018825 (Wang et al., 2013), acc MN105127 (Zhang et al. 2019); *L. burdigalensis* acc NC_029426 (Hissinger et al. 2015); L. oxyrrhis acc NC_029425 (Hissinger et al. 2015); *L. idus* acc CM038646 (unpublished), acc MT584106 (unpublished); *R. rutilus* acc NC_098671 (unpublished), acc OM736800 (unpublished) *A. brama* acc MT410936 (Margaryan et al., 2021), acc NC_020356 (Imoto et al. 2013); *B. bjoerkna* acc NC_020355 (Imoto et al. 2013); *V. melanops* acc NC_031539 (unpublished); *P. phoxinus* acc NC_06871; *P. parva* acc KJ135626 (Xu et al. 2016), acc NC_015614 (Chen et al. 2012); *G. gobio* acc AB239596 (Saitoh et al. 2006), acc OX442399 (unpublished). Photo: B. Delling ©.

Our mitogenome-based phylogenetic analysis (Figure 2) confirms the monophyly of the genus *Leuciscus.* It also supports the placement of other *Leuciscus* species available in GenBank within the genus *Leuciscus* as *L. leuciscus* has been defined as the type species of the genus *Leuciscus.* It also discriminates well Asian (being paraphyletic with *L. merzbacheri, L. baicalensis,* and *L. waleckii*) from European species. In these, Southwestern French species (*L. burdigalensis* and *L. oxyrrhis*) are distinct from septentrional ones (*L. leuciscus* and *L. idus*).

## Discussion and conclusion

In the present study, we determined for the first time the complete mitochondrial genome of *L. leuciscus*. Our mtDNA is highly similar to those of other *Leuciscus* species (Hu et al. 2015; Meng et al. 2023; Wang et al. 2013, 2016) with comparable arrangement of genes. Our phylogenetic analysis corroborates previous work on mitochondrial and nuclear data (Costedoat et al. 2006; Perea et al. 2010; Geiger et al. 2014; Meng et al. 2023). The low divergence between *L. burdigalensis* and *L. oxyrrhis* pinpoints the need for a taxonomical revision of this genus in Southwestern-France. The close relationship between *L. leuciscus* and *L. idus* is due to ancient introgression events during the Pleistocene (Costedoat et al. 2006). This new mitochondrial genome sequence associated with a voucher specimen provides a reliable basis for the identification of this species for further studies, such as the environmental DNA of metabarcoding.

## Supplementary Material

Figure S1.tiff

## Data Availability

The genome sequence data of *Leuciscus leuciscus* that support the findings of this study is openly available in GenBank of NCBI (https://www.ncbi.nlm.nih.gov/) under the accession no. PQ059854. The associated BioProject, Bio-Sample and SRA numbers were PRJNA1150490, SAMN43286068 and SRR30327722, respectively. All the information concerning the specimen can be found in the Swedish Museum of Natural History database (https://artedi.nrm.se/nrmfish/find.php?Category=catalogNumber&Precision=%3D&FormData=55438&Ordering=default&Extent=All&MaxRecs=10&Verbosity=Full&Map=Map&Submit=Submit).

## References

[CIT0001] Berg LS. 1949. Freshwater fishes of the U.S.S.R. and adjacent contries. Israel Program for Scientific Translations, Jerusalem. Vol. 2, 467–926.

[CIT712394] Blanchard E. 1866. Les poissons des eaux douces de la France, Baillère, Paris. p. 1–656.

[CIT0002] Caffrey JM, Hayden B, Walsh T. 2007. Dace (*Leuciscus leuciscus* L.): an Invasive Fish Species in Ireland. Irish Freshwater Fishereies Ecology and Management No. 5. Central Fisheries Board, Dublin, Ireland

[CIT0003] Chen T, Shi YR, You P. 2012. Sequence and analysis of complete mitochondrial genome of *Pseudorasbora parva*. Acta Zootaxonomica Sinica. 37(1):10–19.

[CIT0004] Costedoat C, Chappaz R, Barascud B, Guillard O, Gilles A. 2006. Heterogeneous colonization pattern of European Cyprinids, as highlighted by the dace complex (Teleostei: cyprinidae). Mol Phylogenet Evol. 41(1):127–148. doi:10.1016/j.ympev.2006.04.022. 16797193

[CIT0005] Cuvier G. 1816. Le Règne Animal distribué d’après son organisation pour servir de base à l’histoire naturelle des animaux et d’introduction à l’anatomie comparée. In Les reptiles, les poissons, les mollusques et les annélides, vol. 2. Belin A. Paris.

[CIT16850988] Cuvier G, Valenciennes A. 1844. Histoire naturelle des poissons., Bertrand, Paris. Vol. 17. p. 1–497.

[CIT59185557] Darriba D, Taboada GL, Doallo R, Posada D. 2012. jModelTest 2: more models, new heuristics and parallel computing. Nat Methods. 9(8):772. doi: 10.1038/nmeth.2109. PMC: 22847109. PMC4594756 22847109

[CIT0006] Denys GPJ, Secci-Petretto G, Gomes dos Santos A. 2020. The complete mitochondrial genome of *Thymallus thymallus* (Linnaeus, 1758) (Actinopterygii, Salmonidae) obtained by long range PCRs and double multiplexing. Cybium. 44(2):91–94.

[CIT21327421] Denys GPJ, Dettai A, Persat H, Poulet N, Keith P. Inventory of Biodiversity Today: New Methods and Discoveries, Nicolas V., London 2024. p. 183–200. ITSE edns New Species of Freshwater Fish in France: Reasons and Impacts for Management.

[CIT0008] Dubut V, Martin JF, Gilles A, Van Houdt J, Chappaz R, Costedoat C. 2009. Isolation and characterization of polymorphic microsatellite loci for the dace complex: *leuciscus leuciscus* (Teleostei: cyprinidae). Mol Ecol Resour. 9(4):1179–1183. doi:10.1111/j.1755-0998.2009.02594.x. 21564868

[CIT28177679] Dybowski BN. 1869. Vorläufige Mittheilungen über die Fischfauna des Ononflusses und des Ingoda in Transbaikalien. Verhandlungen der K.-K. Zoologisch-Botanischen Gesellschaft in Wien. V. 19:945–958.

[CIT28265] Dybowski BN. 1874. Die Fische des Baical-Wassersystemes. Verhandlungen der K.-K.. Zoologisch-Botanischen Gesellschaft in Wien. V. 24:383–394.

[CIT0009] Ericson PGP, Zuccon D, Nyström, Edmark V. 2020. A DNA key to Swedish vertebrates – final report. Reports from the Swedish Museum of Natural History. 2020:2.

[CIT0010] Fricke R, Eschmeyer WN, Van der Laan R. 2024. Eschmeyer’s catalog of fishes: genera, species, references. Electronic version accessed 11 jun 2024. http://researcharchive.calacademy.org/research/ichthyology/catalog/fishcatmain.asp.

[CIT0011] Geiger MF, Herder F, Monaghan MT, Almada V, Barbieri R, Bariche M, Berrebi P, Bohlen J, Casal‐Lopez M, Delmastro GB, et al. 2014. Spatial heterogeneity in the Mediterranean Biodiversity Hotspot affects barcoding accuracy of its freshwater fishes. Mol Ecol Resour. 14(6):1210–1221. doi:10.1111/1755-0998.12257. 24690331

[CIT5489346] Heckel JJ. 1837. Ichthyologische Beiträge zu den Familien der Cottoiden, Scorpaenoiden, Gobioiden und Cyprinoiden. Annalen Des Wiener Museums Der Naturgeschichte. V. 2(1):143–164.

[CIT0012] Hinsinger D, Debruyne R, Thomas M, Denys G, Mennesson MI, Utage J, Dettai A. 2015. Fishing for barcodes in the Torrent: from COI to complete mitogenomes on NGS platforms. DNA Barcodes. 3(1):170–186. doi:10.1515/dna-2015-0019.

[CIT0013] Hu S, Niu J, Xie P, Liu C, Karjan A, Wang F, Ma X. 2015. The complete mitochondrial genome of *Leuciscus leuciscus baicalensis* (Cypriniformes: cyprinidae). Mitochondrial DNA Part B. 26(5):751–752. doi:10.3109/19401736.2013.848353. 24460156

[CIT0014] Imoto JM, Saitoh K, Sasaki T, Yonezawa T, Adachi J, Kartavtsev YP, Miya M, Nishida M, Hanzawa N. 2013. Phylogeny and biogeography of highly diverged freshwater fish species (Leuciscinae, Cyprinidae, Teleostei) inferred from mitochondrial genome analysis. Gene. 514(2):112–124. doi:10.1016/j.gene.2012.10.019. 23174367

[CIT0015] Lelek A. 1987. The freshwater fishes of Europe. Threatened fishes of Europe. Vol. 9. Wiesbaden.

[CIT0016] Aula-Verlag, Katoh K, Misawa K, Kuma KI, Miyata T. 2002. MAFFT: a novel method for rapid multiple sequence alignment based on fast Fourier transform. Nucleic Acids Res. 30(14):3059–3066. doi:10.1093/nar/gkf436. 12136088 PMC135756

[CIT0017] Kocher TD, Thomas WK, Meyer A, Edwards SV, Pääbo S, Villablanca FX, Wilson AC. 1989. Dynamics of mitochondrial DNA evolution in animals: amplification and sequencing with conserved primers. Proc Natl Acad Sci U S A. 86(16):6196–6200. doi:10.1073/pnas.86.16.6196. 2762322 PMC297804

[CIT0018] Kottelat M. 1997. An heuristic checklist of the freshwater fishes of Europe (exlusive of former USSR), with an introduction for non-systematists and comments on nomenclature and conservation. Biologia Section Zool. 52(suppl):1–271.

[CIT0019] Kottelat M. 2006. Fishes of Mongolia: a check-list of the fishes known to occur in Mongolia with comments on systematics and nomenclature. Washington, DC: The World Bank.

[CIT1474567] Kottelat M, Freyhof J. 2007. Handbook of European freshwater fishes., Publications Kottelat. p. 1–646.

[CIT1502240] La Blanchère Ha. 1873. Sur une Vandoise nouvelle déterminé dans les eaux du Rouergue (Squalius oxyrrhis, La Bl.). Comptes Rendus Hebdomadaires Des Séances De L’académie Des Sciences. v. 76:662–665.

[CIT0020] Margaryan A, Noer CL, Richter SR, Restrup ME, Bülow‐Hansen JL, Leerhøi F, Langkjær EMR, Gopalakrishnan S, Carøe C, Gilbert MTP, et al. 2021. Mitochondrial genomes of Danish vertebrate species generated for the national DNA reference database, DNAmark. Environ DNA. 3(2):472–480. doi:10.1002/edn3.138.

[CIT0021] Meng W, Li L, Yuan X, Zhou Y. 2023. The complete mitochondrial genome of *Leuciscus merzbacheri* (Cypriniformes: cyprinidae). Mitochondrial DNA Part B Resour. 8(3):414–417. doi:10.1080/23802359.2023.2189496. PMC10035939 36969326

[CIT0022] Miller MA, Pfeiffer W, Schwartz T. 2010. Creating the CIPRES Science Gateway for inference of large phylogenetic trees. In 2010 gateway computing environments workshop (GCE) (pp. 1–8). Ieee. doi:10.1109/GCE.2010.5676129.

[CIT0023] Miya M, Nishida M. 2015. The mitogenomic contributions to molecular phylogenetics and evolution of fishes: a 15-year retrospect. Ichthyol Res. 62(1):29–71. doi:10.1007/s10228-014-0440-9.

[CIT0024] Perea S, Böhme M, Zupancic P, Freyhof J, Sanda R, Ozuluğ M, Abdoli A, Doadrio I. 2010. Phylogenetic relationships and biogeographical patterns in Circum-Mediterranean subfamily Leuciscinae (Teleostei, Cyprinidae) inferred from both mitochondrial and nuclear data. BMC Evol Biol. 10(1):265. doi:10.1186/1471-2148-10-265. 20807419 PMC2940817

[CIT0025] Saitoh K, Sado T, Mayden RL, Hanzawa N, Nakamura K, Nishida M, Miya M. 2006. Mitogenomic evolution and interrelationships of the Cypriniformes (Actinopterygii: ostariophysi): the first evidence toward resolution of higher-level relationships of the world’s largest freshwater fish clade based on 59 whole mitogenome sequences. J Mol Evol. 63(6):826–841. doi:10.1007/s00239-005-0293-y. 17086453

[CIT0026] Schroeter J, Maloy A, Rees C, Bartron M. 2020. Fish mitochondrial genome sequencing: expanding genetic resources to support species detection and biodiversity monitoring using environmental DNA. Conservation Genet Resour. 12(3):433–446. doi:10.1007/s12686-019-01111-0.

[CIT0027] Stamatakis A. 2014. RAxML version 8: a tool for phylogenetic analysis and post-analysis of large phylogenies. Bioinformatics. 30(9):1312–1313. doi:10.1093/bioinformatics/btu033. 24451623 PMC3998144

[CIT0028] Tan M, Armbruster J. 2018. Phylogenetic classification of extant genera of fishes of the order Cypriniformes (Teleostei: ostariophysi). Zootaxa. 4476(1):6–39. doi:10.11646/zootaxa.4476.1.4. 30313339

[CIT9964497] Temmick, CJ, Schlegel H. 1846. Fauna Japonica, sive descriptio animalium, quae in itinere per Japoniam suscepto annis 1823-1830 collegit, notis, observationibus et adumbrationibus illustravit Ph. Fr. de Siebold., Sielbold, P.F., Lugduni Batavorum [Leiden], 173, 269.

[CIT0029] Wang B, Ji P, Xu J, Sun J, Yang J, Xu P, Sun X. 2013. Complete mitochondrial genome of *Leuciscus waleckii* (Cypriniformes: cyprinidae: *leuciscus*). Mitochondrial DNA Part B Resour. 24(2):126–128. doi:10.3109/19401736.2012.731406. 23098409

[CIT0030] Wang F, Niu J, Hu S, Xie P, Liu C, Li H, Karjan A, Ma X. 2016. The complete mitochondrial genome of *Leuciscus idus* (Cypriniformes: cyprinidae). Mitochondrial DNA Part A DNA Mapp Seq Anal. 27(1):104–105. doi:10.3109/19401736.2013.873924. 24438297

[CIT0031] Wiley EO, Mayden RL. 2000. The evolutionary species concept. In A Debate, Wheeler QD & Meier R, editors. Species Concepts and Phylogenetic Theory.). New York: Columbia University Press, p. 70–89.

[CIT0032] Xu W, Geng LW, Xu M, Tong GX, Jiang HF. 2016. Mitochondrial DNA sequence of *Pseudorasbora parva* (Cyprinidae: gobioninae). Mitochondrial DNA Part A DNA Mapp Seq Anal. 27(1):416–417. doi:10.3109/19401736.2014.898284. 24621220

[CIT0033] Zhang H, Xu D, Shi L, Dou H, Sha W. 2019. The complete mitochondrial genome of Amur ide (*Leuciscus waleckii waleckii*). Mitochondrial DNA Part B Resour. 4(2):3702–3704. doi:10.1080/23802359.2019.1679679. PMC7707412 33366151

[CIT0034] Zheng S, Poczai P, Hyvönen J, Tang J, Amiryousefi A. 2020. Chloroplot: an online program for the versatile plotting of organelle genome. Front Genet. 11:576124. doi:10.3389/fgene.2020.576124. 33101394 PMC7545089

[CIT0035] Zhu T, Sato Y, Sado T, Miya M, Iwasaki W. 2023. MitoFish, MitoAnnotator, and MiFish Pipeline: updates in ten years. Mol Biol Evol. 40(3) msad035. doi:10.1093/molbev/msad035. 36857197 PMC9989731

[CIT73873219] Zugmayer E. 1912. LXXIV.— On a new genus of Cyprinoid fishes from high Asia. Annals and Magazine of Natural History. 9(54):682–682. doi:10.1080/00222931208693184.

