# Supplementary figures and images for: The complete mitochondrial genome of the common dace Leuciscus leuciscus (Teleostei: Leuciscidae)
Source: Mitochondrial DNA B Resour. 2025 Feb 2;10(3):173–8. doi: 10.1080/23802359.2025.2460779 (PMC11792123; doi:10.1080/23802359.2025.2460779)

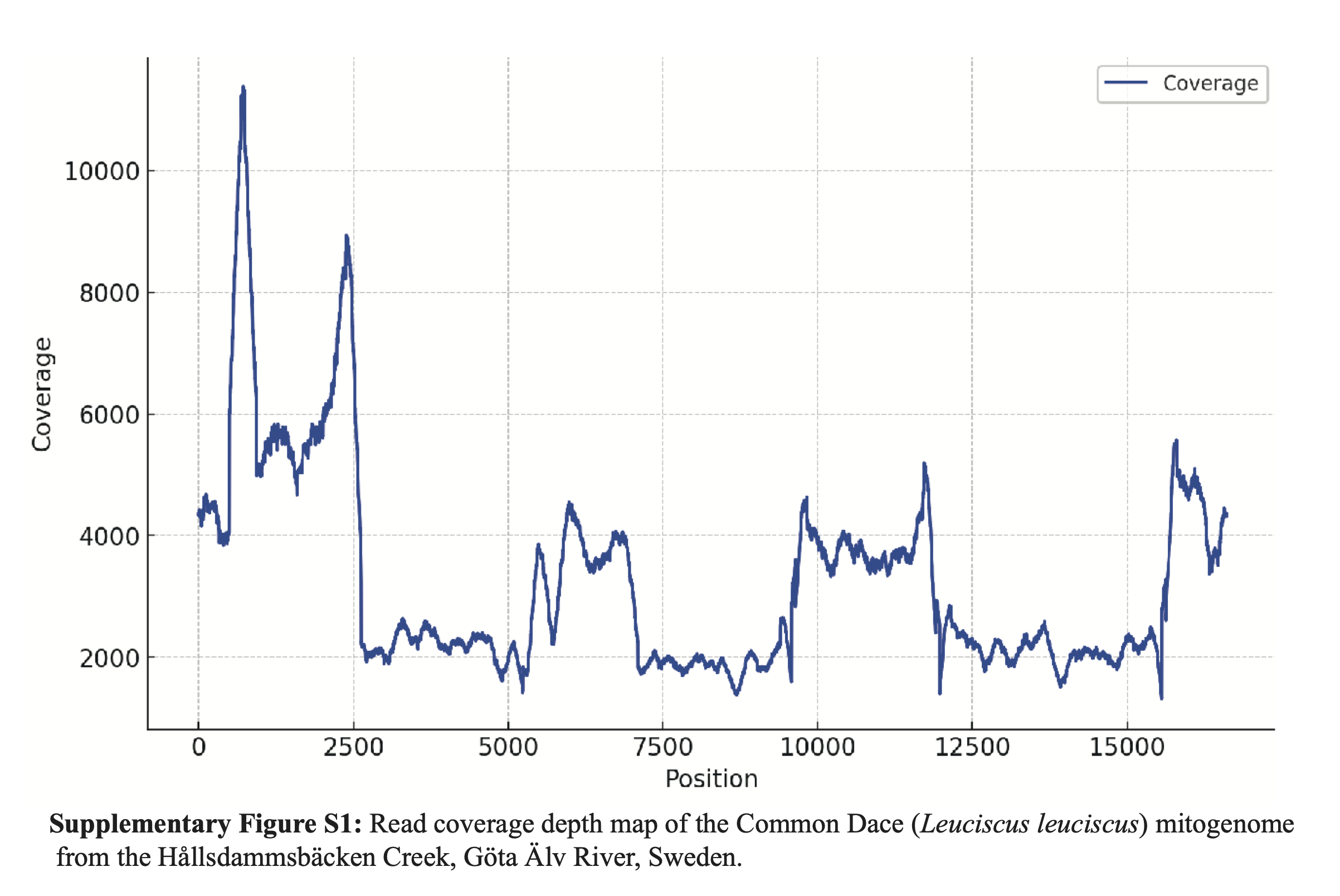

Supplement: Figure S1.tiff [file TMDN_A_2460779_SM6854.tiff]
